# Quantitative assessment of symptomatic intracranial atherosclerosis and lenticulostriate arteries in recent stroke patients using whole-brain high-resolution cardiovascular magnetic resonance imaging

**DOI:** 10.1186/s12968-018-0465-8

**Published:** 2018-06-07

**Authors:** Mengnan Wang, Fang Wu, Yujiao Yang, Huijuan Miao, Zhaoyang Fan, Xunming Ji, Debiao Li, Xiuhai Guo, Qi Yang

**Affiliations:** 1 Department of Neurology, Xuanwu Hospital, Capital Medical University, Beijing, 100053 China; 2 Department of Radiology, Xuanwu Hospital, Capital Medical University, Beijing, 100053 China; 3 Department of Neurology, Sanbo Brain Hospital, Capital Medical University, Beijing, 100093 China; 4 Biomedical Imaging Research Institute, Cedars Sinai Medical Center, Los Angeles, CA 90048 USA; 5 Department of Neurosurgery, Xuanwu Hospital, Capital Medical University, Beijing, 100053 China

**Keywords:** Intracranial atherosclerotic stenosis, High-resolution cardiovascular magnetic resonance imaging, Stroke, Lenticulostriate arteries

## Abstract

**Background:**

It has been shown that intracranial atherosclerotic stenosis (ICAS) has heterogeneous features in terms of plaque instability and vascular remodeling. Therefore, quantitative information on the changes of intracranial atherosclerosis and lenticulostriate arteries (LSAs) may potentially improve understanding of the pathophysiological mechanisms underlying stroke and may guide the treatment and work-up strategies. Our present study aimed to use a novel whole-brain high-resolution cardiovascular magnetic resonance imaging (WB-HRCMR) to assess both ICAS plaques and LSAs in recent stroke patients.

**Methods:**

Twenty-nine symptomatic and 23 asymptomatic ICAS patients were enrolled in this study from Jan 2015 through Sep 2017 and all patients underwent WB-HRCMR. Intracranial atherosclerotic plaque burden, plaque enhancement volume, plaque enhancement index, as well as the number and length of LSAs were evaluated in two groups. Enhancement index was calculated as follows: ([Signal intensity (SI)_plaque_/SI_normal wall_ on post-contrast imaging] − [SI_plaque_/SI_normal wall_ on matched pre-contrast imaging])/(SI_plaque_ / SI_normal wall_ on matched pre-contrast imaging). Logistic regression analysis was used to investigate the independent high risk plaque and LSAs features associated with stroke.

**Results:**

Symptomatic ICAS patients exhibited larger enhancement plaque volume (20.70 ± 3.07 mm^3^ vs. 6.71 ± 1.87 mm^3^ *P* = 0.001) and higher enhancement index (0.44 ± 0.08 vs. 0.09 ± 0.06 *P* = 0.001) compared with the asymptomatic ICAS. The average length of LSAs in symptomatic ICAS (20.95 ± 0.87 mm) was shorter than in asymptomatic ICAS (24.04 ± 0.95 mm) (*P* = 0.02). Regression analysis showed that the enhancement index (100.43, 95% CI − 4.02-2510.96; *P* = 0.005) and the average length of LSAs (0.80, 95% CI − 0.65-0.99; *P* = 0.036) were independent factors for predicting of stroke.

**Conclusion:**

WB-HRCMR enabled the comprehensive quantitative evaluation of intracranial atherosclerotic lesions and perforating arteries. Symptomatic ICAS had distinct plaque characteristics and shorter LSA length compared with asymptomatic ICAS.

## Background

Intracranial atherosclerotic stenosis (ICAS) is one of the most common causes of ischemic stroke, which is associated with high morbidity and mortality rates in Asian countries [1–4]. The initial and follow-up assessment of stroke patients rely mostly upon the evaluation of luminal stenosis via several methods, including transcranial Doppler (TCD), computed tomography angiography (CTA) and magnetic resonance angiography (MRA) [5–8]. Recently, high-resolution cardiovascular magnetic resonance imaging (HR-CMR) has been used to directly depict intracranial vessel wall plaques [9, 10]. Two-dimensional imaging techniques were commonly used for HR-CMR to assess intracranial atherosclerotic plaque morphology and plaque composition [11–13]. However, limited spatial temporal resolution hampered its application in quantitative measurement of vessel wall dimensions and visualization of lenticulostriate arteries (LSAs). Several studies have demonstrated that flow-sensitive black blood magnetic resonance angiography (FSBB-MRA) based on 3D gradient-echo sequence can be specifically used to visualize LSAs [14–16]. Our recent studies have demonstrated the feasibility of whole-brain high-resolution magnetic resonance imaging (WB-HRCMR),which enables combined evaluation of plaque and LSAs in one image setting [17, 18]. Thus, in this study, we aimed to use WB-HRCMR to quantitatively investigate different features of plaque and LSAs in symptomatic versus asymptomatic ICAS groups.

## Methods

### Study population

From January 2015 to September 2017, consecutive symptomatic and asymptomatic ICAS patients who were admitted to or visit the Department of Neurology of our hospital were consecutively recruited. The inclusion criteria: (1) age 18–80 years old; (2) symptomatic ICAS referred to first time acute ischemic stroke in the middle cerebral artery (MCA) territory identified by diffusion weighted imaging (DWI) performed within 72 h of symptom onset, and asymptomatic ICAS referred to patients who were diagnosed with other diseases without history of stroke but had MCA stenosis confirmed on image screening; (3) All enrolled subjects had moderate (stenosis: 50–69%) or severe (stenosis: 70–99%) MCA stenosis, confirmed by MRA, CTA, or digital subtraction angiography. The exclusion criteria included: (1) DWI with lacunar infarction: cerebral infarction in LSAs territory involving less than two layers or the diameter of the infarction < 15 mm; (2) coexistent ipsilateral internal carotid stenosis; (3) preexisting conditions such as vasculitis, moyamoya disease, dissection, reversible cerebral vasoconstriction syndrome (RCVS); (4) evidence of cardioembolism (e.g., arterial fibrillation, mechanical prosthetic valve disease, sick sinus syndrome, dilated cardiomyopathy). All patients underwent WB-HRCMR within 2 weeks of symptom onset. Informed consent was obtained from all participants, and all protocols were approved by the Institutional Review Board.

### WB-HRCMR

All patients underwent WB-HRCMR with a 3-Tesla system (Magnetom Verio; Siemens Healthineers, Erlangen, Germany) and a standard 32-channel head coil. WB-HRCMR was performed at both pre-contrast and post-contrast states by using a 3D T1-weighted whole-brain vessel wall CMR technique known as inversion-recovery (IR) prepared SPACE (Sampling Perfection with Application-optimized Contrast using different flip angle Evolutions) [17, 18], with the following parameters: TR/TE = 900/15 ms; field of view = 170 × 170 mm^2^; 240 slices with slice thickness of 0.53 mm; voxel size = 0.53 × 0.53 × 0.53 mm^3^; scan time = 8 min. The CMR contrast agent, gadopentetate dimeglumine (Magnevist; Schering, Berlin, Germany), was injected through an antecubital vein (0.1 mmol per kilogram of body weight), and WB-HRCMR was repeated 5 min after injection was performed.

### WB-HRCMR image analysis

Evaluation of WB-HRCMR was conducted in consensus by two experienced neuroradiologists blinded to the patient’s clinical details. Commercial software (Vessel Analysis, Oak Medical Imaging Technologies, Inc.) with 3D multi-planar reformation and region-of-interest (ROI) signal measurement functionalities was used for quantitative analysis. A plaque was defined as thickening of the vessel wall using its adjacent proximal, distal, or contralateral vessel segment as a reference. A culprit plaque was defined as (1) the only lesion within the vascular vicinity of the stroke or (2) the most stenotic lesion when multiple plaques were present within the same vascular territory of the stroke. The vessel area (VA) and lumen area were measured by manually tracing vessel and lumen boundaries. The difference between VA and lumen area was the wall area (WA). Stenosis degree was defined as (1-lesion lumen area/reference lumen area) × 100%. The remodeling index (RI) was calculated as the ratio of the lesion VA to the reference VA. The wall area index was defined as the ratio of the lesion WA to the reference WA. And the plaque burden was calculated as WA/VA × 100%. The mean signal intensity (SI) values of culprit plaques and reference vessel wall were measured on pre- and post-contrast WB-HRCMR images.

**Fig. 1:**
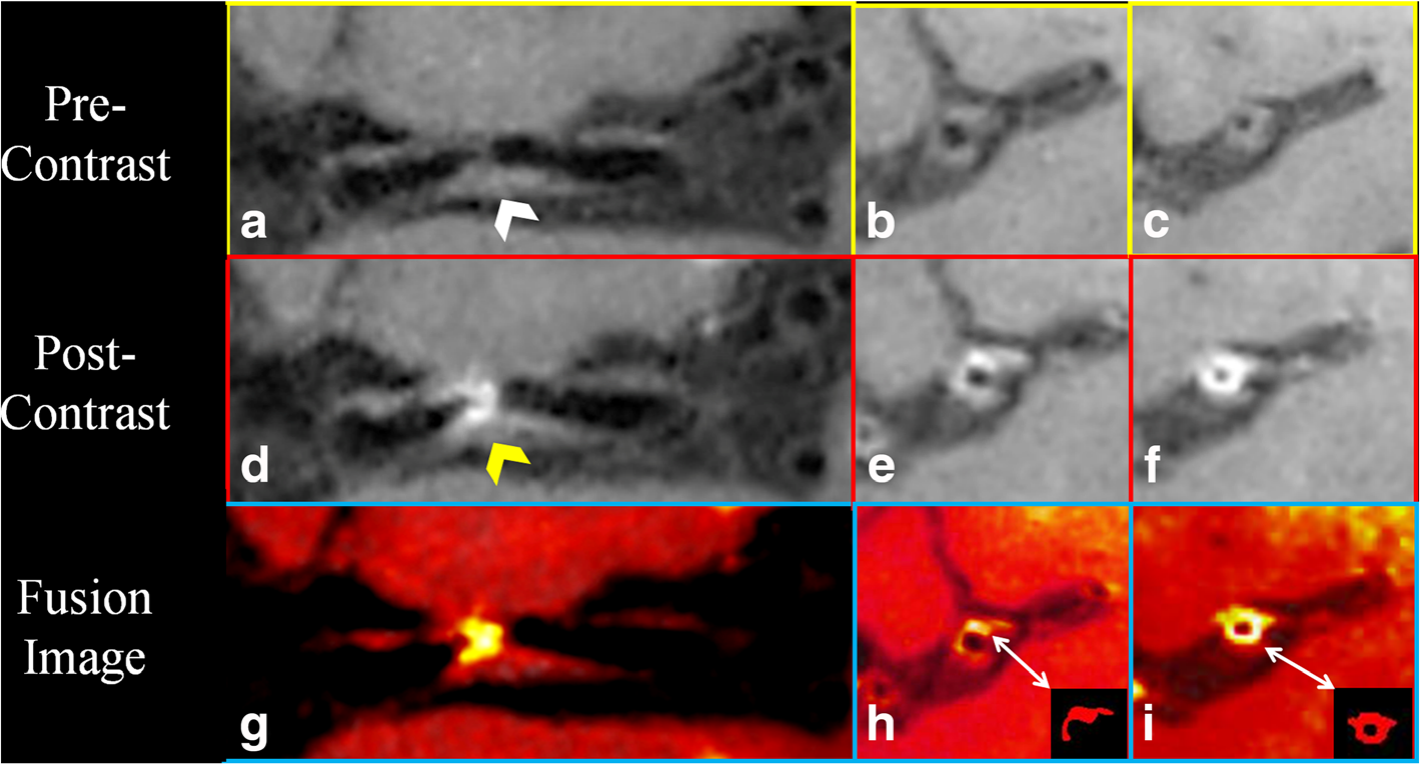
Pre-contrast coronal and cross-sectional whole brain high resolution cardiovascular magnetic resonance (WB-HRCMR) images showed diffused plaque (**a**-**c**, white arrow) located on middle cerebral artery (MCA). Partial enhancement of the plaque was observed (**d**-**f**, yellow arrow). The enhancement plaque area was segmented through fusion images (**h**, **i**). The plaque enhanced volume was 11.02mm^3^

Pre- and post-contrast WB-HRCMR were first co-registered and two-dimensional short-axis images were then generated for the measurement of MCA plaque enhancement. Care was taken to ensure that the short-axis views of the plaque were perpendicular to the M1 segment of MCA. Fusion image of pre- and post-contrast WB-HRCMR have been utilized for the segmentation of the enhanced area of plaque and the enhancement volume was then calculated (Fig. 1). Enhancement index was calculated as follows: ([SI_plaque_/SI_normal wall_ on postcontrast imaging] − [SI_plaque_/SI_normal wall_ on matched precontrast imaging])/(SI_plaque_ / SI_normal wall_ on matched precontrast imaging).

LSAs images were generated using five to six slices of minimum intensity projection (MinIP) in coronal direction with 10–15 mm thickness on pre-contrast WB-HRCMR. LSA branches longer than 5 mm were traced and analyzed by using these images [14]. When LSA branches less than 5 mm from the MCA origin, each branch was counted and measured separately, because more than 70% of branches were found to originate from common trunks [19].

### Statistical analysis

All quantitative data were expressed as means ± standard deviations. Categorical variables were analyzed using Chi-square test and continuous variables were compared using t-test between the two groups. A logistic regression analysis with the method of enter stepwise was used to look for independent predictors of stroke. A *P*-value of less than 0.05 indicated statistical significance. All statistical analyzes were performed by using commercial software (SPSS 22.0, International Business Machnines, Armonk, New York, USA).

## Results

### Patient characteristics

One hundred and one patients were consecutively recruited in the study and forty-nine patients were excluded from analysis due to poor image quality (*N* = 5), < 50% MCA stenosis (*N* = 4), evidence of cardio embolism (*N* = 7), patients with other etiologies (*N* = 13), and patients with lacunar infarction (*N* = 20). The remaining 52 patients were enrolled of which 29 were symptomatic. The demographic data was illustrated in Table 1. No statistically significant differences in patient demographics and the main clinical characteristics were found.

**Table 1:** Demographic in symptomatic and asymptomatic ICAS patients

	Symptomatic ICAS (*N* = 29)	Asymptomatic ICAS (*N* = 23)	*P* value
Age (mean ± SD, years)	46.00 ± 2.32	50.30 ± 2.36	0.397
Male No. (%)	20(69)	13 (56.5)	0.632
Body mass index (mean ± SD, kg/cm^2^)	24.86 ± 1.08	24.75 ± 0.74	0.939
Hypertension No. (%)	12(41.4)	11(47.8)	0.780
Diabetes No. (%)	4(13.8)	4(17.4)	1
Hyperlipidemia No (%)	10(34.5)	9(39.1)	0.778
Smoker No. (%)	15(51.7)	11(47.8)	0.477
LDL-c (mean ± SD, mmol/L)	2.23 ± 0.14	2.32 ± 0.15	0.274
HDL-c (mean ± SD, mmol/L)	1.04 ± 0.05	1.08 ± 0.08	0.203
Triglycerides (mean ± SD, mmol/L)	1.45 ± 0.12	1.43 ± 0.17	0.380
Total cholesterol (mean ± SD, mmol/L)	3.80 ± 0.17	3.67 ± 0.22	0.451

*LDL-c* Low Density Lipoprotein-cholesterol, *HDL-c* High Density Lipoprotein-cholesterol

### ICAS plaque location

A total of seventy-nine ICAS plaques were observed. In symptomatic ICAS group, 29 (61.7%) plaques were found in MCA and 18 (38.3%) were in intracranial ICA. In asymptomatic ICAS group, 23 (71.8%) plaques were detected in MCA, 9 (28.2%) were in intracranial ICA. No statistically significant differences in MCA plaque distribution were found (*P* = 0.469) between the two groups. All culprit plaques in MCA were included for the final analysis.

### ICAS plaque characteristics

**Fig. 2:**
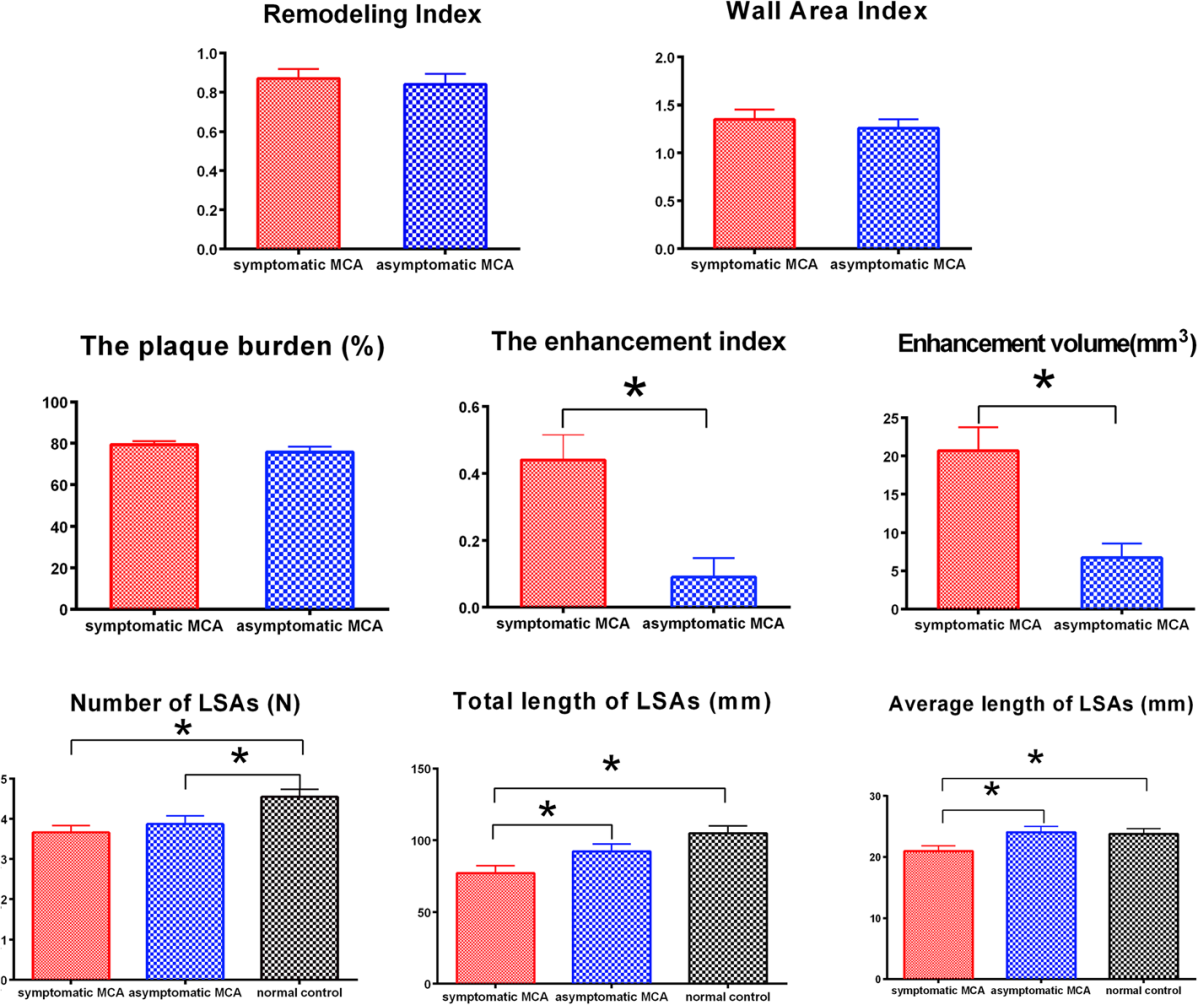
Comparison of remodeling index, wall area index, plaque burden, enhancement index, enhanced volume, number of lenticulostriate arteries (LSAs) and length of LSAs in symptomatic and asymptomatic MCA groups

**Fig. 3:**
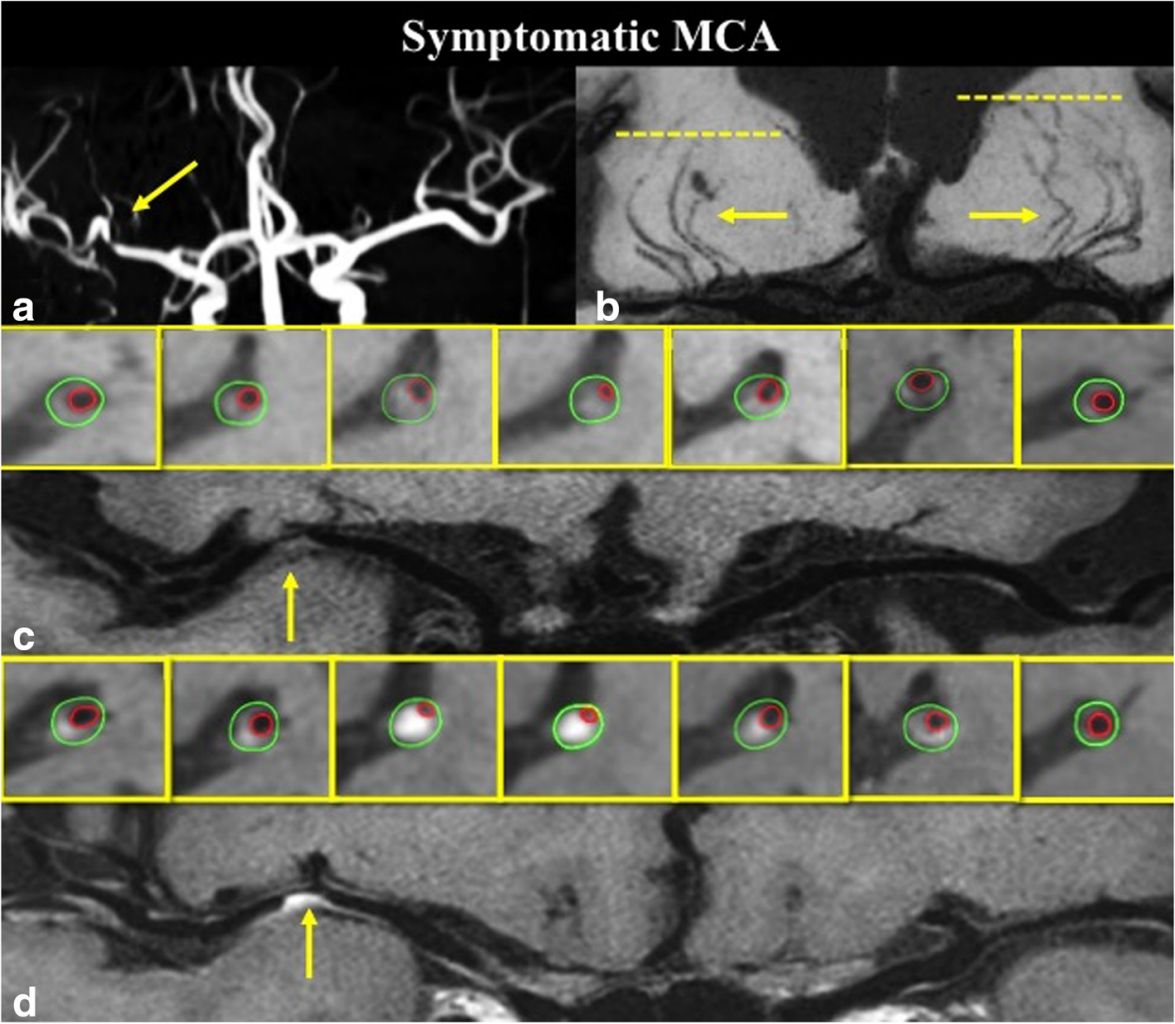
A 61 years old symptomatic ICAS patient with severe stenosis on right MCA (**a**), coronal MinIP revealed the decrease of right LSA branches compared to the left side (**b**); pre-contrast curved WB-HRCMR and cross-sectional images showed a plaque (**c**, arrow) on the MCA wall; Post-contrast WB-HRCMR showed extensive enhanced plaque volume which can be measured on corresponding cross-sectional images

**Fig. 4:**
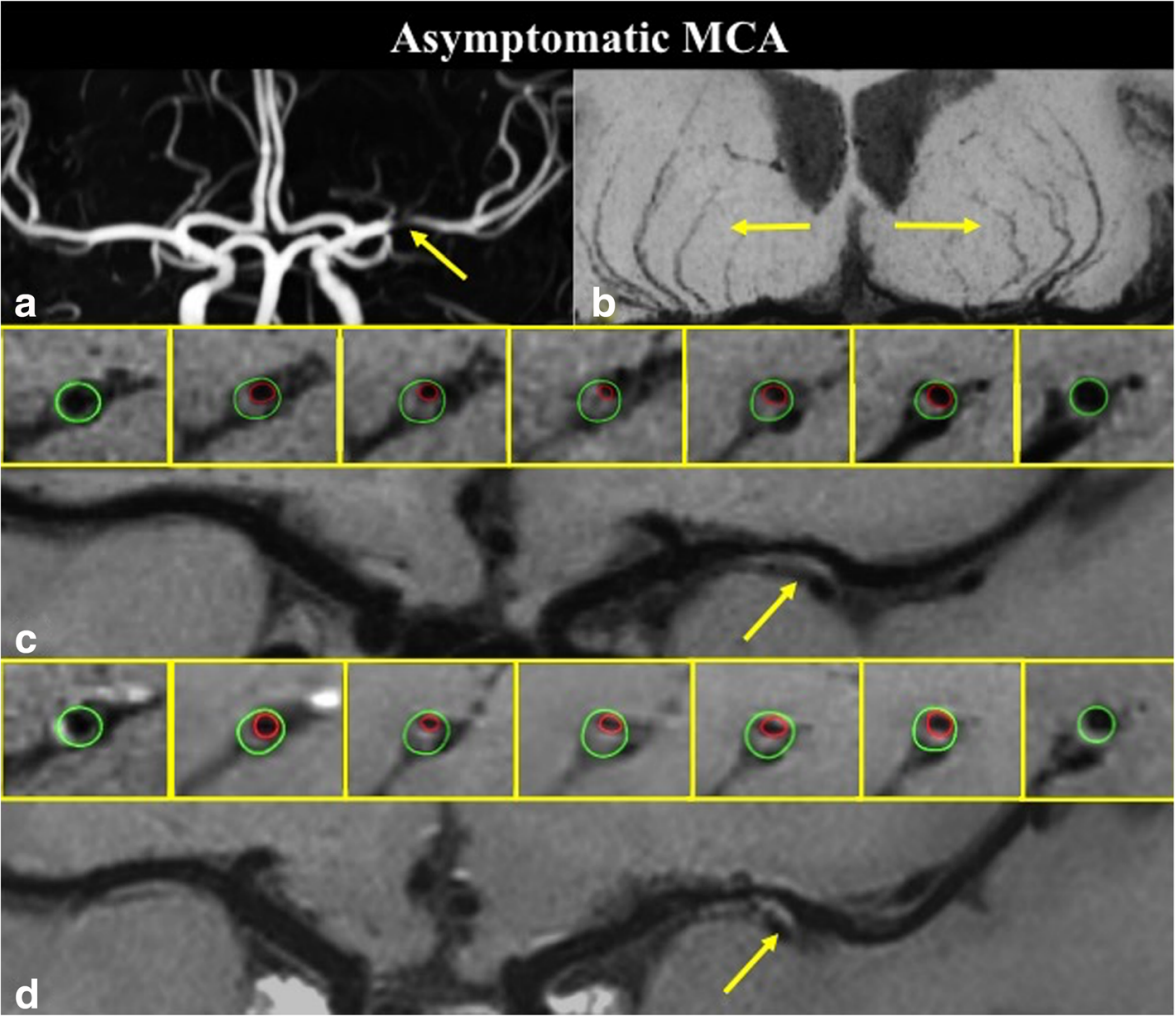
A 65 years old asymptomatic ICAS patient with severe stenosis on left MCA (**a**), coronal minimum intensity projection (MinIP) revealed symmetrical LSAs of the left and right hemispheres (**b**); pre-contrast curved WB-HRCMR and cross-sectional images showed a plaque (**c**, arrow) on the ventral and inferior side of MCA wall; post-contrast WB-HRCMR showed no enhancement

A total of fifty-two MCA plaques were included for the final analysis. The degrees of stenoses, RI, WA, and plaque burden did not differ significantly between two groups. Symptomatic MCA demonstrated greater plaque enhancement, including larger enhancement volume (20.70 ± 3.07 mm^3^ vs. 6.71 ± 1.87 mm^3^, *P* = 0.001) and higher enhancement index (0.44 ± 0.08 vs. 0.09 ± 0.06, *P* = 0.001) (Fig. 2). Two representative cases of symptomatic and asymptomatic MCA are presented in Figs. 3 and 4.

### The LSAs features

In order to compare the several features of LSAs, twenty age-and sex-matched healthy subjects were included as normal controls. The mean number of LSAs was 3.65 ± 0.18 in symptomatic group, 3.87 ± 0.21 in asymptomatic group and 4.55 ± 0.19 on normal controls, respectively. There was significant difference between symptomatic group and normal controls (*P* = 0.002), and asymptomatic group also had statistical differences in LSAs branches compared with normal controls (*P* = 0.020). Symptomatic group had significant shorter total length of LSAs than normal controls (*P* < 0.001) but no difference was found between asymptomatic and normal groups (*P* = 0.111). The symptomatic group had shorter average length than both the asymptomatic groups (*P* = 0.02) and the normal controls (*P* = 0.034). Table 2 summarizes detailed characteristics of the two groups.

**Table 2:** Plaque features and logistic regression analyses in symptomatic and asymptomatic ICAS

	Symptomatic ICAS (N = 29)	Asymptomatic ICAS (N = 23)	*P* value	Univariate Analysis OR(95%CI)	*P* value	Multivariable Analysis OR(95%CI)	*P* value
Degree of stenoses (mean ± SD, %)	63.60 ± 2.80	63.94 ± 3.02	0.936	1.01 (0.99–1.04)	0.424	–	–
Remodeling index (mean ± SD,)	0.87 ± 0.05	0.84 ± 0.06	0.868	1.57(0.18–13.64)	0.681	–	–
Wall area index (mean ± SD)	1.35 ± 0.10	1.26 ± 0.09	0.517	1.46(0.47–4.50)	0.509	–	–
Plaque burden (mean ± SD, %)	79.39 ± 1.64	75.71 ± 2.68	0.227	1.03(0.98–1.09)	0.238	–	–
Enhancement volume (mean ± SD, mm^3^)	20.70 ± 3.07	6.71 ± 1.87	0.001	1.02(1.02–1.18)	0.005	–	–
Enhancement index (mean ± SD)	0.44 ± 0.08	0.09 ± 0.06	0.001	23.65(2.63–213.02)	0.005	100.43 (4.02–2510.96)	0.005
Number of LSA (mean ± SD, N)	3.65 ± 0.18	3.87 ± 0.21	0.433	0.80(0.44–1.41)	0.425	–	–
Total length of LSA (mean ± SD,mm)	77.12 ± 5.16	92.16 ± 5.15	0.047	0.98(0.96–1.00)	0.054	–	–
Average length of LSA(mean ± SD,mm)	20.95 ± 0.87	24.04 ± 0.95	0.020	0.86(0.76–0.98)	0.027	0.80(0.65–0.99)	0.036

*OR* Odds ratio, *CI* Confidence interval

### Multivariate analysis

In a logistic regression analysis, the higher enhancement index and shorter average length of LSAs were independently associated with stroke. Odds ratios for enhancement index and average length of LSAs were 100.43 and 0.80 (95% confidence interval 4.02–2510.96 and 0.65–0.99; *P* = 0.005 and 0.036) respectively.

## Discussion

In this study, we found that symptomatic MCA plaques exhibited a higher enhancement index and larger enhancement volume than the asymptomatic group. Furthermore, a significant reduction in the average number and length of LSAs in symptomatic ICAS groups was also found. To the best of our knowledge, this is the first study using WB-HRCMR to quantitatively explore the intracranial high risk plaque characteristics and LSA features in one imaging setting in ICAS patients.

Although variable refocusing flip angle sequences have been the most extensively studied 3D techniques for intracranial vessel wall imaging to date, it is still associated with inadequate suppression of cerebrospinal fluid (CSF) signals and limited field of view. Some lesions may be missed, especially in the more distally vessels. This may cause an underestimation of the true intracranial plaque burden. WB-HRCMR technique allows for whole brain coverage, relatively high and isotropic spatial resolution, and more importantly, remarkable suppression of CSF and enhanced T1 contrast weighting. It enables the measurement of total intracranial plaque burden, plaque morphology and perforating arteries together.

Previous studies found that enhancement of an intracranial atherosclerotic plaque is associated with a recent ischemic event, and is independent of plaque thickness [20–24]. However, in most studies, the extent of plaque enhancement was not quantitatively measured [21, 24–26] and qualitative methods have been used to categorize the degree of plaque enhancement by comparing the enhancement of plaque and the pituitary on MR [25]. Our findings are in line with the these studies, however, with a step forward quantitative method. We registered and fused the pre- and post-contrast WB-HRCMR images and contour the enhanced plaque volume, accordingly. Thus, WB-HRCMR enables more accurate measurements of intracranial atherosclerosis plaques characteristics, such as enhancement index and the enhancement volume. We observed that symptomatic MCAs had higher enhancement index and larger enhanced volume of intracranial atherosclerosis plaques.

Recent studies have demonstrated that FSBB-MRA can be used to visualize LSAs [14–16, 27]. Our recent studies proved the feasibility of using whole-brain intracranial vessel wall imaging to depict LSA branches [17, 18]. The mean number of LSA branches on normal controls in our study was 4.55, which is consistent with Okuchi’s and Kang’s previous studies [15, 28]. Compared with normal controls, symptomatic MCAs had a significant decrease in the number and the length of LSAs.

There were several limitations in our study. First, this is an observational study and longitudinal studies are warranted to investigate and expound on the usage of WB-HRCMR in the prediction of stroke outcome and the risk of recurrent stroke. Secondly, the mechanism of plaque enhancement remains unclear and there is no pathological validation of the intracranial plaque vulnerability. Finally, due to the relatively limited spatial resolution used, it is difficult to evaluate the distal small perforating arteries. Partial volume effect of the volume measurement can be overcome by further optimizing imaging parameters or applying with higher field strength.

## Conclusions

WB-HRCMR enabled the comprehensive quantitative evaluation of vessel wall lesions and the LSAs in stroke patients. Symptomatic MCAs have larger enhanced plaque volume, higher enhancement plaque index, and shorter length of LSAs compared with asymptomatic MCAs.

## Abbreviations

CSF: Cerebrospinal fluid
CTA: Computed tomography angiography
DWI: Diffusion weighted imaging
FSBB-MRA: Flow-sensitive black blood magnetic resonance angiography
HDL: High density lipoprotein
HR-CMR: High-resolution cardiovascular magnetic resonance imaging
ICAS: Intracranial atherosclerotic stenosis
IR: Inversion-recovery
LDL: Low density lipoprotein
LSA: Lenticulostriate artery
MCA: Middle cerebral artery
MinIP: Minimum intensity projection
MRA: Magnetic resonance angiography
RCVS: Reversible cerebral vasoconstriction syndrome
RI: Remodel index
ROI: Region-of-interest
SI: Signal intensity
SPACE: Sampling Perfection with Application-optimized Contrast using different flip angle Evolutions
TCD: Transcranial Doppler
VA: Vessel area
WA: Wall area
WB-HRCMR: Whole-brain high-resolution cardiovascular magnetic resonance
